# Supplementary material for: Krüppel-like Factor 5 Plays an Important Role in the Pathogenesis of Chronic Pancreatitis
Source: Cancers (Basel). 2023 Nov 15;15(22):5427. doi: 10.3390/cancers15225427 (PMC10670257; doi:10.3390/cancers15225427)
Supplement: Supplementary file 1 [file cancers-15-05427-s001.zip › cancers-2659702-supplementary.pdf]

Supplementary Figure S1.

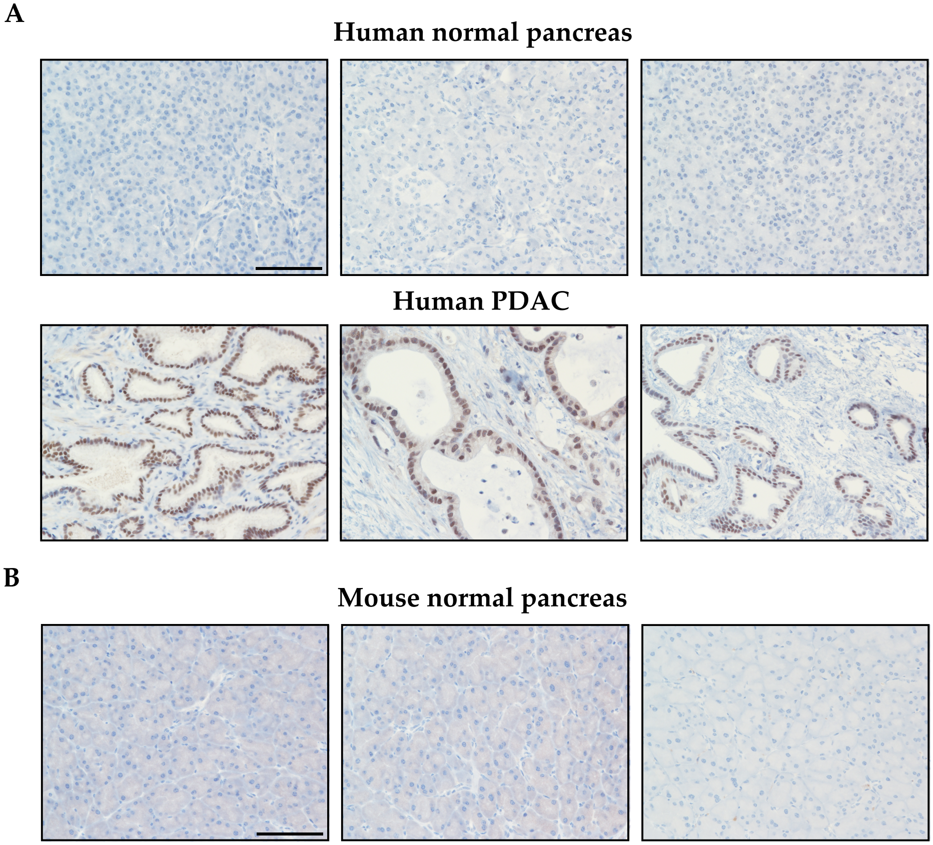

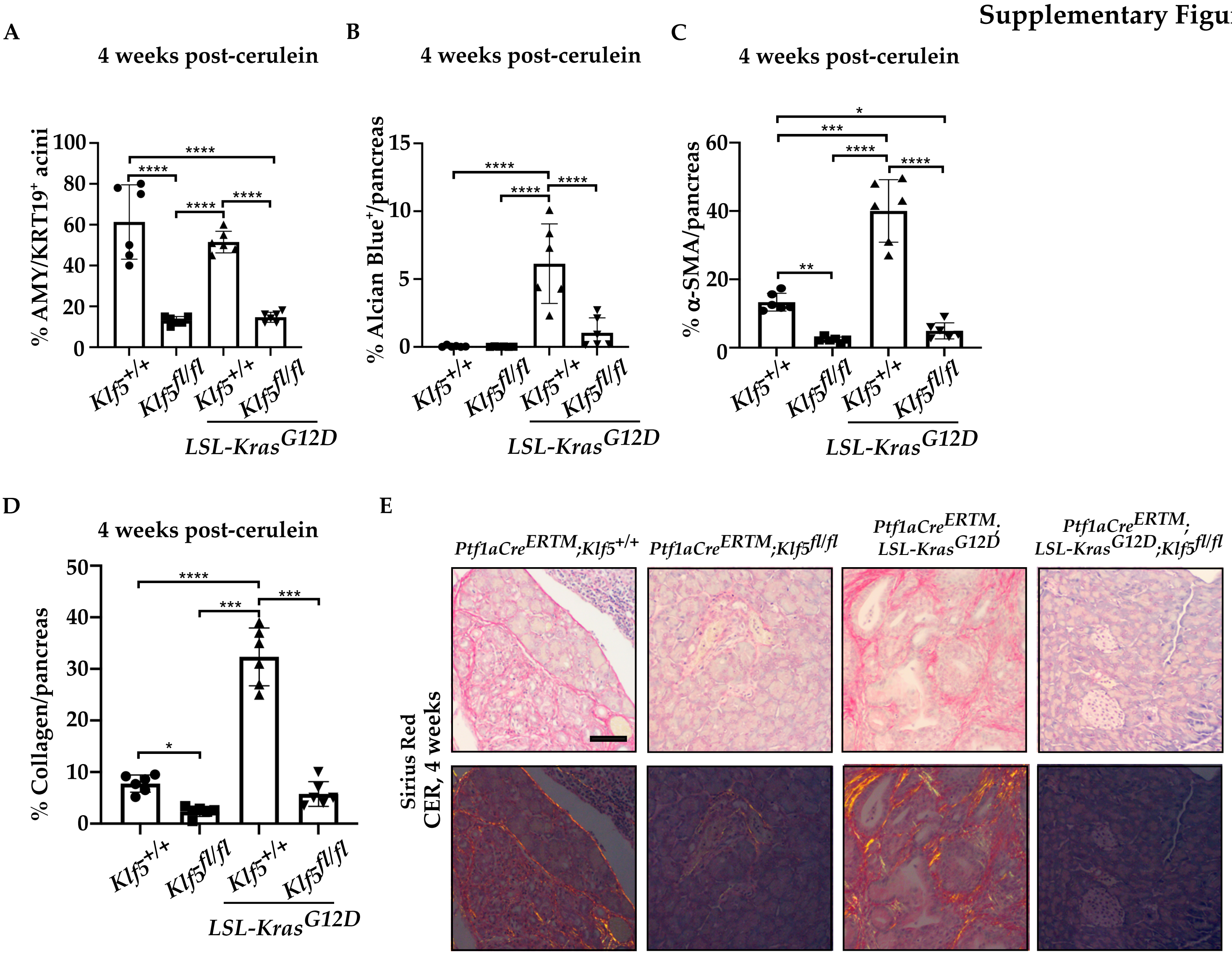
A
4 weeks post-cerulein
% AMY/KRT19+ acini
0
20
40
60
80
100
****
****
****
****
Klf5+/+
Klf5fl/fl
Klf5+/+
Klf5fl/fl
LSL-KrasG12D
B
4 weeks post-cerulein
% Alcian Blue+/pancreas
0
5
10
15
****
****
****
Klf5+/+
Klf5fl/fl
Klf5+/+
Klf5fl/fl
LSL-KrasG12D
C
4 weeks post-cerulein
% α-SMA/pancreas
0
20
40
60
*
***
****
****
**
Klf5+/+
Klf5fl/fl
Klf5+/+
Klf5fl/fl
LSL-KrasG12D
D
4 weeks post-cerulein
% Collagen/pancreas
0
10
20
30
40
50
****
***
***
*
Klf5+/+
Klf5fl/fl
Klf5+/+
Klf5fl/fl
LSL-KrasG12D
E
Ptf1aCreERTM;Klf5+/+
Ptf1aCreERTM;Klf5fl/fl
Ptf1aCreERTM;
LSL-KrasG12D
Ptf1aCreERTM;
LSL-KrasG12D;Klf5fl/fl
Sirius Red
CER, 4 weeks

Supplementary Figure S3.

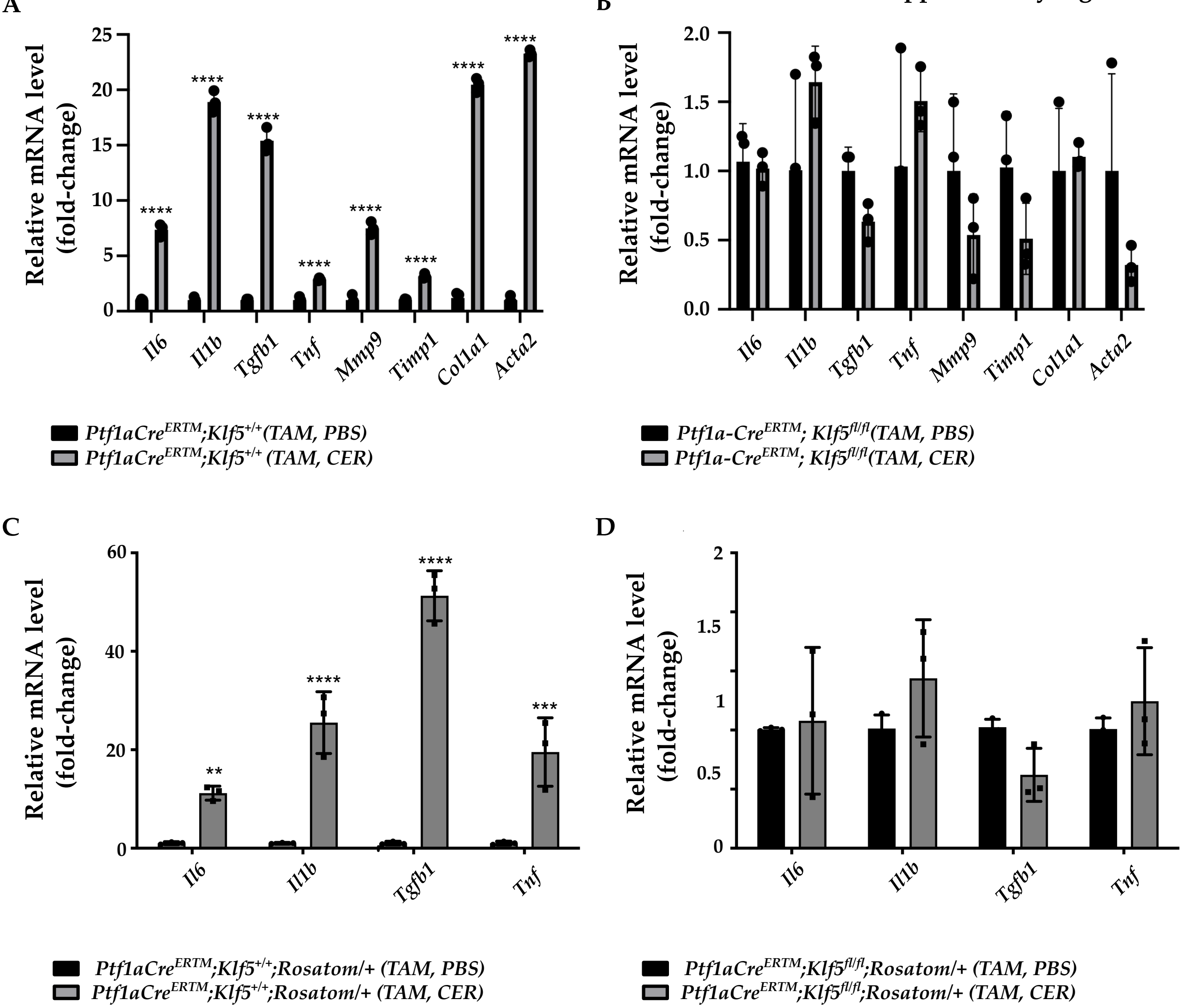

Supplementary Figure S1. Representative IHC images of KLF5 stain in human and mouse pancreas. (A) Negative stain of KLF5 in normal human pancreas (top panel) and positive stain of KLF5 in human PDAC. **(B)** Negative stain of KLF5 in normal mouse pancreas (specifically in pancreatic acinar cells). Scale bars = 200 μm.

Supplementary Figure S2. Genetic inactivation of *Klf5* in vivo suppresses ADM, PanIN, and pancreatic stellate cells activation and fibrosis. (A) Quantification of Amylase/KRT19 positive acini. **(B)** Quantification of Alcian Blue positive area. **(C)** Quantification of αSMA positive area. **(D)** Quantification of collagen positive area. *P<0.05, **P<0.01, ***P<0.001, and ****P<0.0001 by One-Way ANOVA test (Data represent mean ± S.D.) **(E)** Representative images of Picro Sirius Red stain of mice of indicated genotypes and treatment. Top panel images were taken using bright field microscopy, and bottom panel images were taken after exposure to polarized light. Scale bar = 50μm.

Supplementary Figure S3. KLF5 regulates the expression of inflammatory and fibrotic markers during chronic pancreatitis. (A) and **(B)** qRT-PCR results of gene expression of *Il6*, *Il1b*, *Tgfb1*, *Tnf*, *Mmp9*, *Timp1*, *Col1a1*, and *Acta2* in the whole pancreatic tissues from mice of indicated genotypes and treatments. ***P<0.01*, ****P<0.001*, *****P<0.000*, by One-Way ANOVA test (Data represent mean ± S.D.). **(C)** and **(D)** qRT-PCR results of gene expression of *Il6*, *Il1b*, *Tgfb1*, and *Tnf* in tdTomato-positive cells originated from pancreatic tissues from mice of indicated genotypes and treatments. **P<0.05*, ***P<0.01* by One-Way ANOVA test (*N* = 3, Data represent mean ± S.D.).
